# Local Electrical Impedance Mapping of the Atria: Conclusions on Substrate Properties and Confounding Factors

**DOI:** 10.3389/fphys.2021.788885

**Published:** 2022-01-24

**Authors:** Laura Anna Unger, Leonie Schicketanz, Tobias Oesterlein, Michael Stritt, Annika Haas, Carmen Martínez Antón, Kerstin Schmidt, Olaf Doessel, Armin Luik

**Affiliations:** ^1^Institute of Biomedical Engineering, Department of Electrical Engineering and Information Technology, Karlsruhe Institute of Technology, Karlsruhe, Germany; ^2^Boston Scientific, Ratingen, Germany; ^3^Medizinische Klinik IV, Städtisches Klinikum Karlsruhe, Academic Teaching Hospital of the University of Freiburg, Karlsruhe, Germany

**Keywords:** local impedance, impedance mapping, atrial substrate, electrophysiology, atrial fibrillation, pulmonary vein isolation, substrate mapping

## Abstract

The treatment of atrial fibrillation and other cardiac arrhythmias as a major cause of cardiovascular hospitalization has remained a challenge predominantly for patients with severely remodeled substrate. Individualized ablation strategies are extremely important both for pulmonary vein isolation and subsequent ablations. Current approaches to identifying arrhythmogenic regions rely on electrogram-based features such as activation time and voltage. Novel technologies now enable clinical assessment of the local impedance as tissue property. Previous studies demonstrated its use for ablation monitoring and indicated its potential to differentiate healthy substrate, scar, and pathological tissue. This study investigates the potential of local electrical impedance-based substrate mapping of the atria for human *in-vivo* data. The presented pipeline for impedance mapping particularly contains options for dealing with undesirable effects originating from cardiac motion, catheter motion, or proximity to other intracardiac devices. Bloodpool impedance was automatically determined as a patient-specific reference. Full-chamber, left atrial impedance maps were drawn up from interpolating the measured impedances to the atrial endocardium. Finally, the origin and magnitude of oscillations of the raw impedance recording were probed into. The most dominant reason for exclusion of impedance samples was the loss of endocardial contact. With median elevations above the bloodpool impedance between 29 and 46 Ω, the impedance within the pulmonary veins significantly exceeded the remaining atrial walls presenting median elevations above the bloodpool impedance between 16 and 20 Ω. Previous ablation lesions were distinguished from their surroundings by a significant drop in local impedance while the corresponding regions did not differ for the control group. The raw impedance was found to oscillate with median amplitudes between 6 and 17 Ω depending on the patient. Oscillations were traced back to an interplay of atrial, ventricular, and respiratory motion. In summary, local impedance measurements demonstrated their capability to distinguish pathological atrial tissue from physiological substrate. Methods to limit the influence of confounding factors that still hinder impedance mapping were presented. Measurements at different frequencies or the combination of multiple electrodes could lead to further improvement. The presented examples indicate that electrogram- and impedance-based substrate mapping have the potential to complement each other toward better patient outcomes in future.

## 1. Introduction

Electrical impedance measurements have a long history in medical diagnosis and treatment (Malmivuo and Plonsey, 1995; Grimnes and Martinsen, 2000). Measuring the complex resistive behavior of different tissues provides insight into the tissue characteristics (Gabriel et al., 1996). In clinical electrophysiology, cardiac arrhythmias are treated by ablating the triggering or promoting cells with radiofrequency energy and monitoring the impedance during energy delivery. The generator impedance is typically measured with a two-electrode setup consisting of the intracardiac ablation electrode and a cutaneous dispersive electrode. The transthoracic impedance of the radiofrequency energy delivery pathway serves as a measure for lesion assessment. Although the generator impedance is capable of sensing impedance differences between bloodpool and tissue contact, the local resolution is very limited and flawed by the bulk impedance of the torso (van Es et al., 2017). The DirectSense^TM^ technology (Boston Scientific, Marlborough, MA) has recently been introduced to overcome this problem. Novel local impedance (LI) measurement capabilities on ablation catheters with all measuring and injecting electrodes at the catheter itself allow to precisely assess catheter–tissue coupling and are more predictive of lesion formation (Sulkin et al., 2018; Das et al., 2021). The technology takes advantage of acute changes in tissue impedance provoked by radiofrequency ablation (Das et al., 2021).

Besides ablation monitoring, impedance measurements can also characterize different types of tissue that differ in their baseline impedance. Experimental analyses of electrical tissue impedance go back to 1996 with extensive characterization of electrical tissue impedance for a wide range of injection frequencies and a diverse set of human tissue samples and other materials (Gabriel et al., 1996). Based on that effect, the DirectSense^TM^ technology can be used as an investigational tool to characterize cardiac tissue by its electrical impedance (Martin et al., 2018; Gunawardene et al., 2019). Acquiring point-by-point intracardiac impedance data allows for compilation of full-chamber impedance maps. However, tissue characteristics are not the only factor influencing the measurement. Catheter-tissue contact, irrigation fluids, catheter motion, blood flow dynamics, and the proximity to other intracardiac devices have to be taken into account. This study analyzes first experiences with the compilation of meaningful atrial full-chamber LI maps while throwing light on both tissue characteristics as well as confounding factors of LI measurements in the atria.

## 2. Materials and Methods

### 2.1. Study Design

This is a retrospective, single-center study which was conducted in accordance with the Declaration of Helsinki. The study was approved by the local ethics committee and all patients provided written informed consent. Patients who received an electrophysiological study and catheter ablation due to atrial fibrillation (AFib) using the RHYTHMIA HDx^TM^ electroanatomical mapping system (Boston Scientific, Malborough, MA, USA) were included into the study. The left atrium (LA) was fully mapped using the IntellaNav MiFi^TM^ OI (Boston Scientific, Malborough, MA, USA) ablation catheter. Magnetic catheter tracking enhanced localization accuracy. After completion of the study, the geometry, mapping data including local activation time (LAT) and bipolar voltage maps, electrograms, and impedance data were exported from the system for retrospective analysis. Preprocessing steps for both impedance and electrogram recordings were followed by detailed analyses of the impedance data.

### 2.2. Electrogram Processing

#### 2.2.1. QRS Intervals

The timing of all R peaks was extracted from the surface ECG. The precordial leads V3 and V4 showed less susceptibility to pacing artifacts from the coronary sinus (CS) catheter than others and were thus selected for R peak detection with the open-source software *ECGdeli* (Pilia et al., 2020). RR intervals were determined as the time difference between two subsequent R peaks.

#### 2.2.2. Pacing

The bipolar electrograms recorded in the CS catheter served as a source for the detection of pacing events emerging from the CS catheter. Peaks in the bipolar electrogram exceeding an amplitude of 200 mV defined pacing events.

### 2.3. Local Impedance Measurements

The DirectSense^TM^ technology allowed for intra-atrial LI recordings with the IntellaNav MiFi^TM^ OI catheter. An alternating current is injected at 14.5 kHz between the distal tip electrode and the proximal ring electrode to create a local electrical field. The electrical properties of the immediate surroundings impact the formation of the electrical field. The potential difference ΔΦ between each of the three mini-electrodes and the distal ring electrode represents a sample measurement of this electrical field that allows to deduce the electrical properties of the immediately surrounding material. The DirectSense^TM^ technology provides the quotient of the potential difference ΔΦ in the numerator and the injected current amplitude in the denominator (Sulkin et al., 2018) which will be termed LI in the following and carries the physical unit Ω. The LI is recorded with a sampling frequency of 20 Hz.

The raw LI recording LI_raw_ is subject to significant oscillations. Hence, two different approaches for postprocessing were chosen which resulted in the moving average LI_movAvg_ and the upper envelope LI_upEn_.

#### 2.3.1. Moving Average

Following the clinical data acquisition, a window of 1.5 s duration was centered at each point in time to calculate the moving average LI_movAvg_ (Sulkin et al., 2018; Das et al., 2021) in order to smooth out the oscillations in LI_raw_. The moving average was motivated by the assumption that it is a clinically established and potentially robust representation of the underlying impedance.

#### 2.3.2. Upper Envelope

As a second approach, the upper envelope of LI_raw_ was obtained by determining the local maxima and subsequent interpolation. Expecting the tissue impedance to be higher than the bloodpool impedance led to the assumption that tissue impedance is reflected best by the maximum values. Local maxima were characterized by a minimum peak prominence of 4 Ω and a minimum peak-to-peak distance equaling half of the mean RR interval to account for LI oscillations caused by ventricular contraction. Local maxima were then temporally interpolated by minimizing the change in slope; therefore, the second derivative as calculated with the discrete Laplacian operator was minimized with the least squares approach. The derived signal will be termed LI_upEn_ in the following.

### 2.4. Bloodpool Impedance

The LI of the bloodpool (LI_bloodpool_) is typically seen as a patient specific reference value and defined as the LI value measured in the LA bloodpool without tissue contact. Since LI_bloodpool_ varies among patients, it was individually determined by extracting LI sequences according to the following criteria:

- The distance between any IntellaNav MiFi^TM^ OI electrode and the endocardial shell is at least *d*.
- The oscillatory amplitude in LI_raw_ is smaller than the median oscillatory amplitude of the patient. For simplicity, the oscillatory amplitude was defined as the difference between the maximum and minimum LI_raw_ within a centered moving window of 1 s duration in this context.
- LI_movAvg_ or LI_upEn_, respectively, <180 Ω in order to exclude sheath artifacts.
- LI_movAvg_ or LI_upEn_, respectively, does not change by more than 5 Ω/s in order to exclude artifacts.

To account for different atrial sizes, the minimal distance *d* between any IntellaNav MiFi^TM^ OI electrode and the endocardial shell was decreased starting from 15 mm in steps of 1 mm until a total of at least 10 s of bloodpool acquisitions accumulated.

The 25 % quartile of all extracted measurements defined the reference value LI_bloodpool_. A bloodpool measure was calculated from both LI_movAvg_ and LI_upEn_ resulting in two values per patient.

### 2.5. Exclusion of Artifacts

As opposed to LAT and voltage mapping, LI mapping is not restricted to one acquisition per atrial cycle since the LI as target property is independent from atrial excitation in the first place (Amorós-Figueras et al., 2018). Therefore, any acquired LI sample can theoretically be taken into account. However, many confounding factors impede the revelation of tissue characteristics from LI measurements. Measures have to be taken to minimize the impact of undesirable influences on the LI. In particular, LI measurements were excluded from any subsequent analysis if they met one or more of the following conditions:

- **Sheath artifacts**: An overlap of the steerable sheath with electrodes of the IntellaNav MiFi^TM^ OI catheter causes an artificial increase of LI. Since little sheath coverage of the proximal ring may result in slightly elevated LI values similar to acquisitions within the pulmonary veins (PVs), optimal sheath detection is a non-trivial task. The combination of two criteria determined the presence of sheath overlaps: (i) LI_raw_ exceeding LI_bloodpool_ + 200 Ω; (ii) changes in LI_raw_ exceeding 400 $\frac{\Omega }{\text{s}}$. In case of (i), the excluded segment was extended in both directions until LI_raw_ first fell below LI_bloodpool_ + 50 Ω again. Each invalid segment was extended by a safety margin of 1 s in both directions. Remaining valid segments shorter than 0.5 s were excluded as well.
- **Loss of wall contact**: Any LI acquisition taken in distances larger than 7 mm to the endocardium was excluded from further processing.
- **Catheter movement**: Fast catheter movements change the influence of irrigation and blood flow on the LI and result in ambiguous assignments to endocardial locations. The moving average of the velocity within windows of 350 ms quantified catheter movement. Average movements faster than 2 $\frac{\text{mm}}{\text{s}}$ were excluded from further processing to focus on stable catheter positions. Each invalid segment was extended by a safety margin of 1 s in both directions. Remaining valid segments shorter than 0.5 s were excluded as well.
- **Proximity to Orion**: For some patients, the impedance map was recorded while the IntellaMap Orion^TM^ catheter was inside the LA. Metal and insulator components of other catheters such as the Orion can strongly influence the LI recording. Therefore, any LI acquisition taken in less than 5 mm distance to the Orion catheter was excluded from further processing. Each invalid segment was extended by a safety margin of 1 s in both directions. Remaining valid segments shorter than 0.5 s were excluded as well.

In summary, three out of four exclusion criteria were based on catheter positioning while one criterion was based on the LI trace itself.

### 2.6. Quantification of LI Oscillations

Oscillations in LI_raw_ occurred for various reasons. The most dominant oscillation frequency correlated with the ventricular and atrial contraction rate. Therefore, RR intervals served as windows to determine the LI oscillation (LI_osci_) as the difference between the maximum and the minimum value of LI_raw_ in the respective RR interval. Only RR intervals with full coverage of valid LI measurements were attributed with an LI_osci_. The median of all attributed LI_osci_ characterized a patient.

### 2.7. Partitioning of the Left Atrium

#### 2.7.1. Anatomical Partitioning

**Figure 1:**
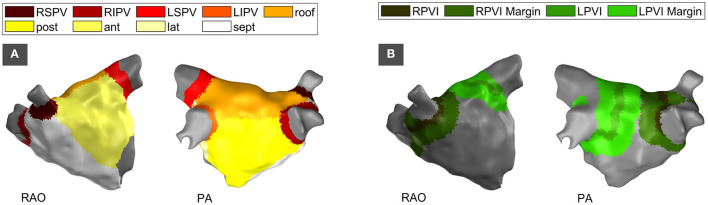
Partitioning of the left atrium by anatomical region **(A)** and by structural region based on the location of PVI lesions **(B)**. Right-anterior-oblique (RAO) and posterior-anterior (PA) perspective. RSPV, right superior pulmonary vein; RIPV, right inferior pulmonary vein; LSPV, left superior pulmonary vein; LIPV, left inferior pulmonary vein; post, posterior; ant, anterior; lat, lateral; sept, septal; RPVI, right pulmonary vein isolation; LPVI, left pulmonary vein isolation; RAO, right-anterior-oblique view; PA, posterior-anterior view.

Each LA geometry was clinically annotated with cutouts for the mitral valve and all PVs. The remaining LA was individually subdivided into the septal wall, the lateral wall, the anterior wall, the posterior wall, the inferior wall, and the PV ostia as color-coded in Figure 1A for an exemplary patient. The full set of the aforementioned regions will be referred to as mapping region. The inner parts of the PVs as removed by cutouts during the electrophysiological study were combined with the PV ostia for the regional comparison of LI recordings. The resulting set of vertices will be termed extended mapping region and includes the entire endocardial shell with the exception of the mitral valve. The mapping region and the extended mapping region underwent the same interpolation process as described below for the purpose of impedance mapping.

#### 2.7.2. Structural Partitioning According to Ipsilateral Pulmonary Vein Isolation

In cases with a history of a previous pulmonary vein isolation (PVI), the spatial position of ipsilateral PVI circles were labeled to assess the capability of LI to distinguish between physiological and pathological or preablated tissue. The location was defined based on the voltage map of the current procedure and—if available—by rigid transformation of the ablation points from the previous procedure into the current coordinate system. For each ipsilateral circle, both the ablation core and a surrounding margin were annotated. The total width of the ablation core was varied from 1 mm to 10 mm in steps of 1 mm during analysis. Each side of the surrounding margin was varied from 1 mm to 20 mm in width. In order to prevent anatomical influences on the analysis of structural LI changes as expected for recording positions within the PVs, any overlap with an inner PV or a PV ostium as defined in the anatomical partitioning was removed from the structurally partitioned regions. Figure 1B displays an exemplary LA with structural labels for ablation cores and surrounding margins. Comparison to the anatomical labels in Figure 1A shows that the PV ostia and the inner PVs are left without label in the structural partitioning.

Patients presenting for de-novo PVI served as control group. Even though the impedance map was recorded before the patients received PVI, the PVI lines were retrospectively annotated according to the location of the ensuing PVI. Extension of the PVI line to the PVI region as well as the definition of the PVI margins was carried out accordingly.

### 2.8. Impedance Mapping

Any valid impedance acquisition was assigned to the closest vertex of the endocardial surface mesh within the mapping area. If one vertex accumulated more than one LI measurement, the set was represented by the 75% quartile. Subsequent nearest neighbor interpolation with a maximum interpolation distance of 5 mm provided LI values for unassigned vertices. Impedance maps were calculated with both LI_movAvg_ and LI_upEn_ as input. The extended mapping area was considered for the regional comparison of LI acquisitions including the PVs while impedance mapping itself was carried out for the mapping area only.

### 2.9. Statistical Analysis

Distributions were tested for normality with the Anderson-Darling test. Non-normal distributions were assumed if the test rejected the null-hypothesis of a normal distribution at a 1 % significance level. Non-normal distributions with unpaired samples were compared with the Mann-Whitney *U*-test. Normally distributed paired data sets were tested with the paired samples *t*-test. The significance level was set to 1 %. Medians and inter-quartile ranges are reported to describe distributions. The central line of all boxplots describes the median while the boxes range from the 25^th^ to the 75^th^ percent quantile. The whiskers extend to the outermost data points that were not considered outliers. The underlying distributions of adjacent boxplots were compared by statistical significance testing as appropriate. Asterisks denote that the null-hypothesis of equal underlying distributions was rejected. The coefficient of determination *R*^2^ was used to describe the relation between two dependent variables.

## 3. Results

### 3.1. Patient Demographics

The study comprised 14 patients (female n = 5). The subjects' age ranged from 41 to 71 years with a mean age of 64 years. Twelve subjects were diagnosed with paroxysmal AFib, two subjects with persistent AFib. Eleven subjects underwent redo PVI while the remaining ones presented for de-novo PVI. During impedance mapping, 5 patients were in sinus rhythm, 4 patients in paced rhythm, 2 patients in AFib, and 3 patients in alternating rhythm including sinus and paced rhythm, AFib, and atrial flutter.

### 3.2. Bloodpool Impedance

**Figure 2:**
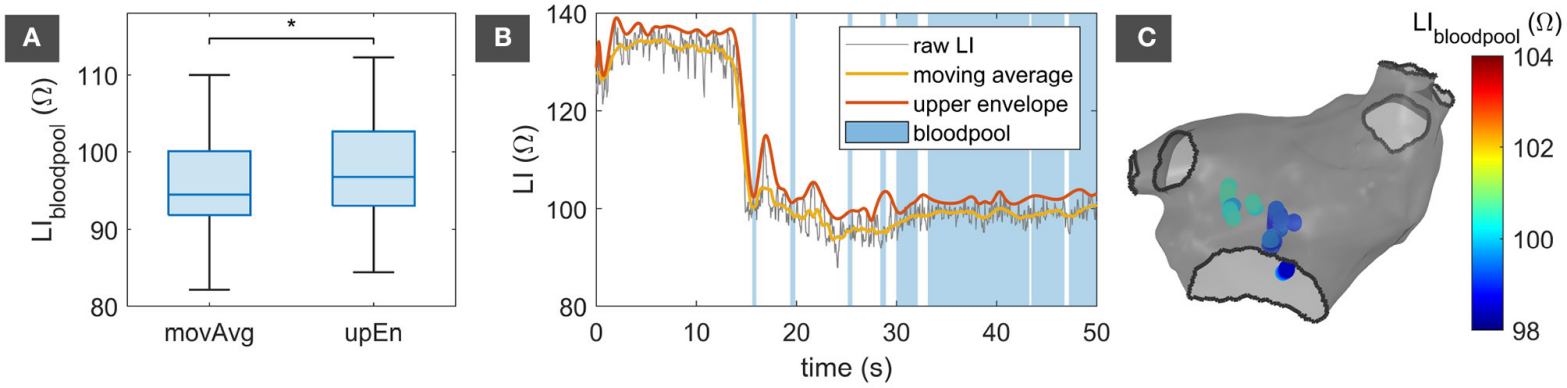
Bloodpool reference impedance. **(A)** Distribution of LI_bloodpool_ retrieved from LI_movAvg_ and LI_upEn_ for all 14 patients. The asterisk (*) denotes statistically significant difference between the distributions. **(B)** Exemplary trace of LI_raw_ with corresponding LI_movAvg_ and LI_upEn_. Samples with valid bloodpool acquisitions are marked in blue. **(C)** LA endocardial shell with spatial location of valid bloodpool acquisitions. movAvg, moving average; upEn, upper envelope; LI, local impedance; LA, left atrium.

Only a slight shift distinguished the reference LI_bloodpool_ depending on whether LI_movAvg_ or LI_upEn_ was provided as the input LI. With a median of 96.8 Ω (inter-quartile range 9.5 Ω) amongst all patients, LI_bloodpool_ retrieved from LI_upEn_ exceeded the median of LI_bloodpool_ retrieved from LI_movAvg_ at 94.6 Ω (inter-quartile range 8.3 Ω). Statistical testing evaluated the distributions as different (*p* = 2·10^−9^). Figure 2A displays the distribution of both LI_bloodpool_ measures for all patients. Any value was within clinically observed ranges and in line with manually noted LI_bloodpool_ values for the individual patients. The deviation between LI_bloodpool_ automatically retrieved from LI_movAvg_ and LI_bloodpool_ retrieved from LI_upEn_ for individual patients ranged from 1.2 to 4.1 Ω with a median of 2.4 Ω.

Figure 2B shows an exemplary excerpt of an LI_raw_ trace overlaid by the corresponding LI_movAvg_ in yellow and LI_upEn_ in orange. Being the upper envelope of LI_raw_, LI_upEn_ exceeds LI_movAvg_. Samples that were taken into account for determining LI_bloodpool_ are marked in blue. LI_raw_ significantly drops at 14 s when the catheter loses endocardial contact. Figure 2C visualizes the locations of acquisition for all LI_bloodpool_ samples with an LI between the 10 % and 90 % quantile color-coded by the respective LI_movAvg_. All acquisitions are located centrally in the LA cavity.

### 3.3. Exclusion of Artifacts

Due to the availability of many measurement points independent from the atrial rhythm in combination with a high probability for artifacts in the LI trace, exclusion criteria were set conservatively. Table 1 gives an overview of timely percentages per exclusion reason. Since an IntellaMap Orion^TM^ catheter was present in less than half of the patients during map acquisition with the IntellaNav MiFi^TM^ OI catheter, both the minimum and median exclusion percentage counted 0.0 %. An increased distance to the endocardium was the most prominent reason for the exclusion of LI samples. The spectrum of percentages with the possibility of an overlapping sheath spread from 1.0 to 32.8 %. The high maximum percentage was associated with cases for which data acquisition continued after completion of mapping when the IntellaNav MiFi^TM^ OI had already been drawn back into the sheath.

**Table 1:** Exclusion of LI measurements.

	**Min (%)**	**Median (%)**	**Max (%)**
Sheath overlap	1.0	8.5	32.8
Catheter movement	5.0	12.2	20.0
Proximity to Orion	0.0	0.0	8.1
Distance to wall	9.2	20.2	46.1

*Minimum, median, and maximum percentage of all patients*.

**Figure 3:**
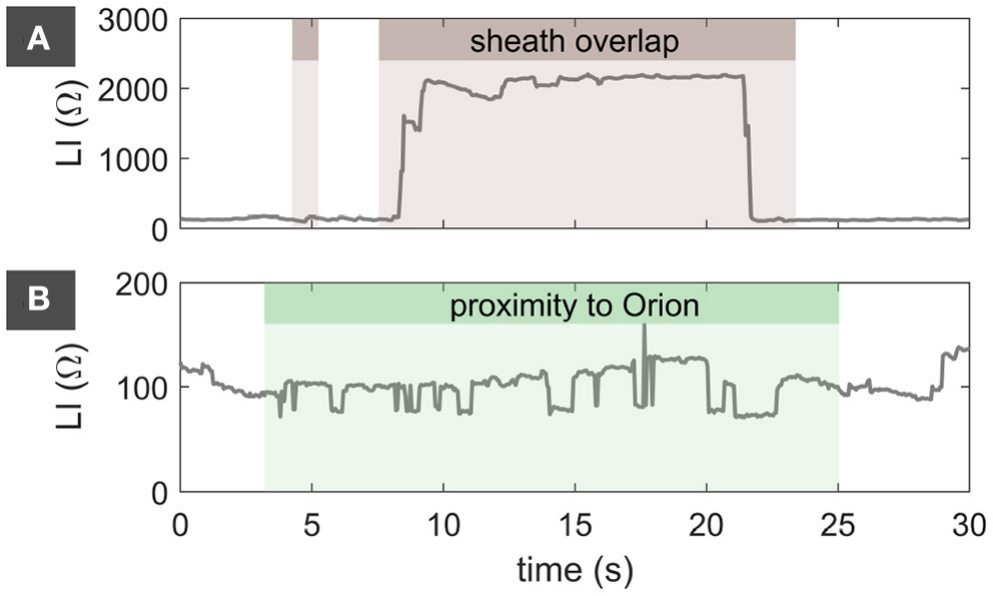
LI_raw_ artifacts attributed to an overlap with the steerable sheath in **(A)** as well as attributed to the proximity to the IntellaMap Orion^TM^ catheter in **(B)**. Brown background marks samples that were excluded due to a suspected overlap with the steerable sheath. Green background marks samples that were excluded due to proximity to the IntellaMap Orion^TM^. LI, local impedance.

Figure 3 presents exemplary traces of characteristic LI traces for artifacts attributed to an overlap with the steerable sheath in panel (A) and attributed to the proximity to the IntellaMap Orion^TM^ in (B). Figure 3A displays the characteristic LI trace for complete sheath coverage. LI_raw_ increases to unphysiological values above 1,000 Ω due to full retraction of the IntellaNav MiFi^TM^ OI into the sheath made from electrically highly resistive material. Other examples with partial coverage of the IntellaNav MiFi^TM^ OI by the sheath were also detected successfully. As opposed to the electrically highly resistive sheath material, other catheters such as the IntellaMap Orion^TM^ are partially composed of metallic materials of low electrical resistance resulting in unphysiologically abrupt drops in LI_raw_ down to 71.0 Ω as depicted in Figure 3B.

### 3.4. Impedance Mapping

#### 3.4.1. LI by Distance to Endocardium

In order to assess a possible causal relationship between the LI measurements and the distance to the endocardial wall, all valid LI acquisitions of all patients were referenced to their individual LI_bloodpool_ by subtraction. A linear regression showed that the LI tends to drop by 1.14 Ω per additional mm distance to the endocardium. However, a low regression coefficient of determination R^2^ = 1 % indicated that the distance to the endocardial wall is only a minor determinant of LI and is joined by a series of other influencing factors that in sum result in the measured LI.

#### 3.4.2. LI by Anatomical Region

**Figure 4:**
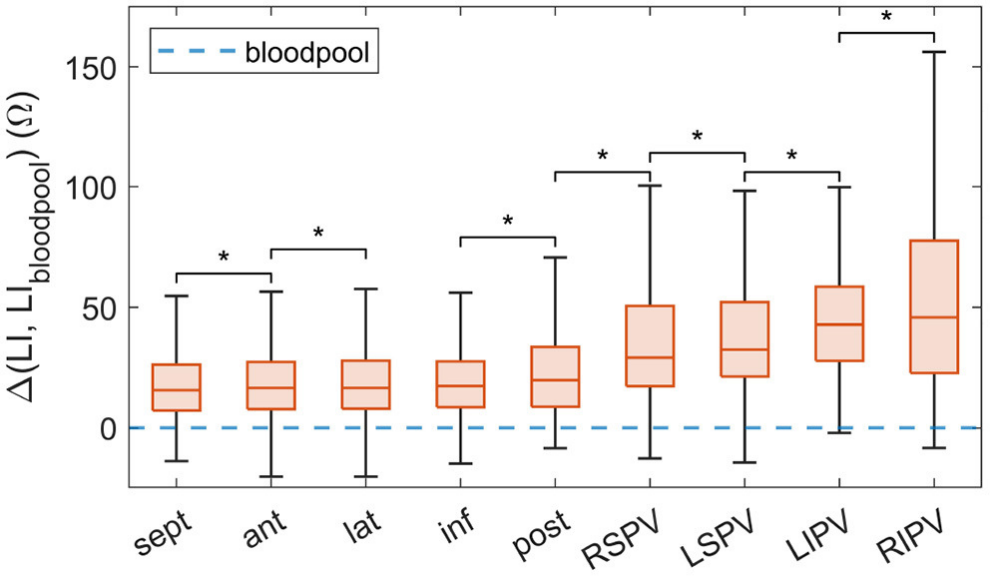
Difference between LI_upEn_ as mapped to the endocardium within the extended mapping region and LI_bloodpool_ combined by anatomical region for all patients. Arranged by ascending median. The virtual LI_bloodpool_ corresponds to 0 Ω. Adjacent distributions which were statistically significantly different are marked with a *. LI, local impedance; sept, septal; ant, anterior; lat, lateral; inf, inferior; post, posterior; RSPV, right superior pulmonary vein; LSPV, left superior pulmonary vein; LIPV, left inferior pulmonary vein; RIPV, right inferior pulmonary vein.

Within individual patients, the distributions of LI measurements at times differed significantly between the anterior, posterior, inferior, septal, and lateral wall. However, a clear trend did not become apparent amongst patients. Referencing all LI acquisitions of all patients to the corresponding reference LI_bloodpool_ allowed for inter-patient statistics as depicted in Figure 4. The impedance ranges measured for the distinct atrial walls were very similar, with a median LI_upEn_ elevation above LI_bloodpool_ between 15.7 and 19.9 Ω and inter-quartile ranges between 19.0 and 24.8 Ω. Both median and inter-quartile range were highest for the posterior wall. The distributions for all four PVs significantly differed from the atrial walls with median LI_upEn_ elevations above LI_bloodpool_ of 29.2 to 45.9 Ω and inter-quartile ranges between 30.8 and 54.8 Ω. The LI within the inferior PVs exceeded the LI within the superior PVs. The Mann-Whitney *U*-test for unpaired samples and a significance level of 1 % yielded that the distribution for any anatomical region was statistically significantly different from any other region with the exception of the lateral and the inferior wall. The *p*-values amongst pairs of the atrial walls were found to exceed the p-values of pairs combining any atrial wall with any PV by several decades.

#### 3.4.3. LI on and Adjacent to Previous PVI Lesion

**Figure 5:**
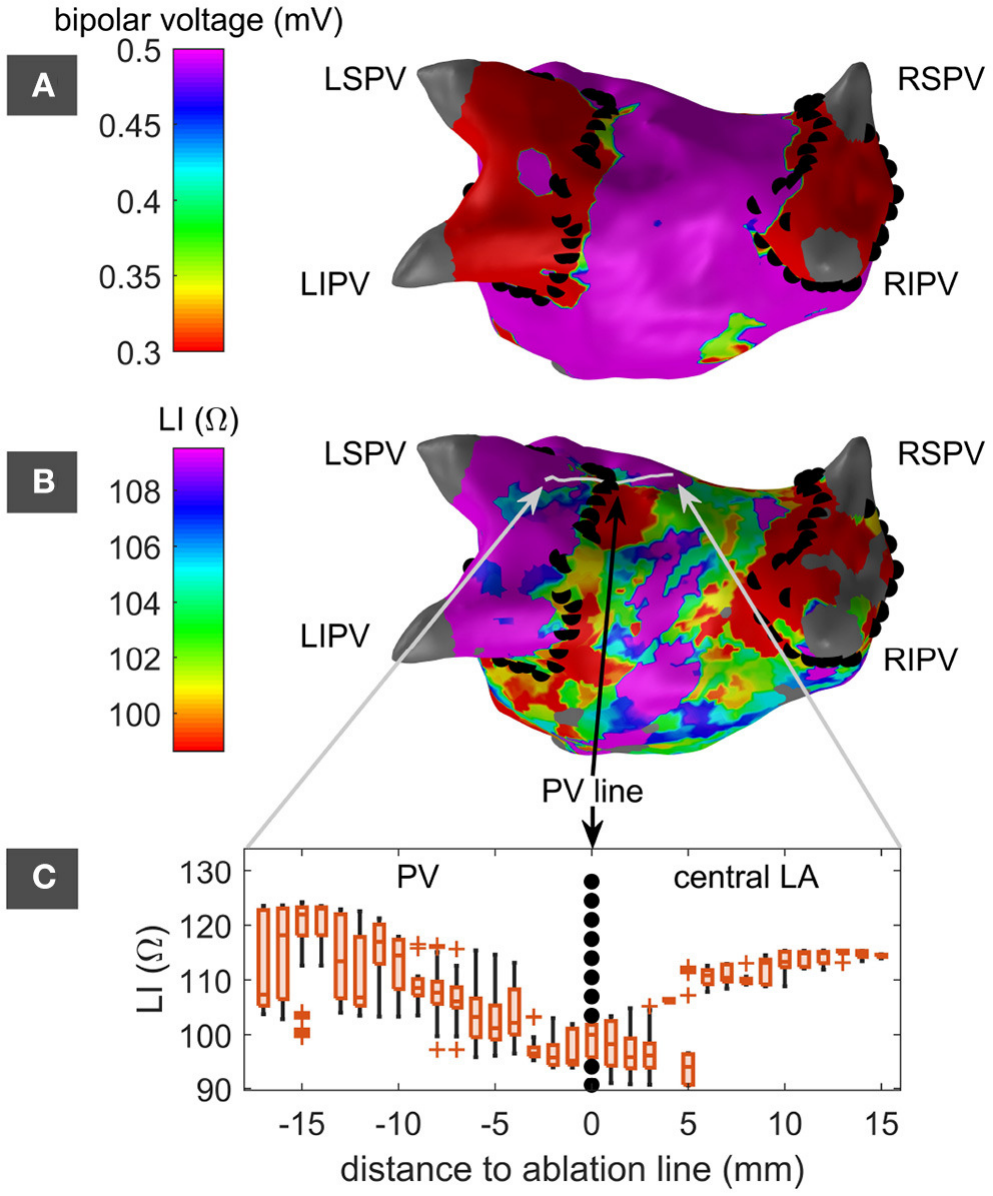
LA in posterior-anterior perspective with color-coded bipolar voltage map **(A)** and LI map **(B)**. Left superior, left inferior, right superior, and right inferior PVs are labeled as LSPV, LIPV, RSPV, and RIPV. PVI ablation points from a previous intervention are marked by black dots. The LI map in panel **(B)** includes a light gray line indicating a path perpendicular to and approximately centered on the previous PVI lesion next to the LSPV. The distribution of LI measurements along this path shows a drop at the previous PVI lesion **(C)**. Adjacent distributions were not tested for statistical significance. LSPV, left superior pulmonary vein; LIPV, left inferior pulmonary vein; RSPV, right superior pulmonary vein; RIPV, right inferior pulmonary vein; PV, pulmonary veins; LA, left atrium; LI, local impedance.

PVI lesions from previous procedures served as regions of known atrial substrate to assess the capabilities of LI to conclude on substrate properties. In a first step, the distribution of LI_upEn_ along a line perpendicular to the PVI lesion from a previous procedure was analyzed. Figures 5A,B show the bipolar voltage map and the LI_upEn_ map for an exemplary patient that had undergone previous PVI. Ablation points from the previous procedure were mapped to the current geometry and are displayed as black dots in Figure 5. The ablation points were found to match the step in the voltage map in Figure 5A indicating the position of the PVI lesion. Any LI acquisition originating from within a tube of 7 mm radius centered on the gray line perpendicular to the PVI lesion in Figure 5B was projected to the gray line and assigned a geodesic distance to the PVI line along the endocardial mesh. Figure 5C summarizes the distribution of LI_upEn_ along the gray line. While LI_upEn_ drops below 100 Ω close to the lesion, it rises above 110 Ω with increasing distance to the previous PVI in both directions (LA roof and left superior PV). Both the variance and the absolute value were higher toward the left superior PV compared to the LA roof.

**Figure 6:**
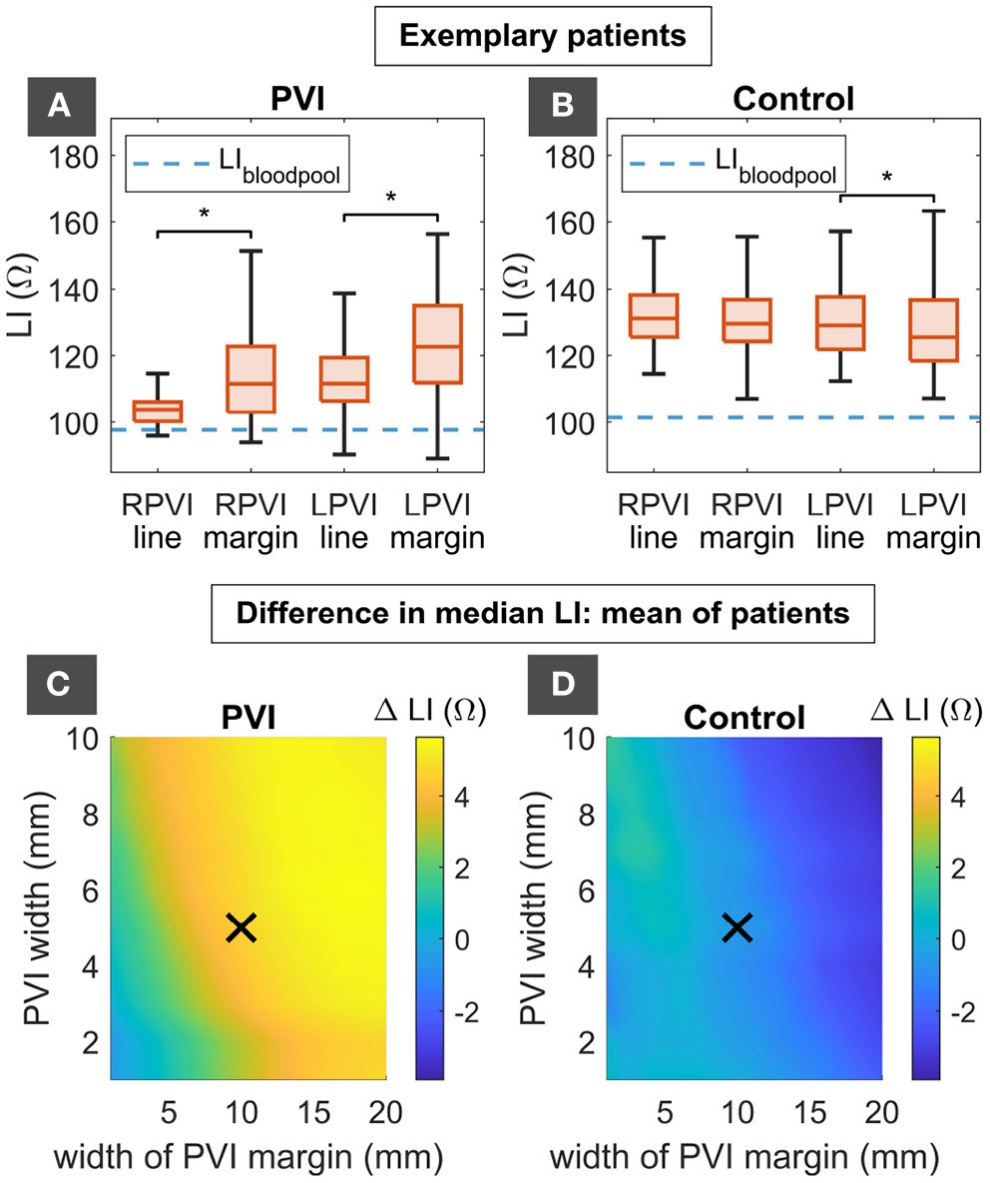
Distribution of LI_upEn_ for an exemplary patient with previous PVI **(A)** and an exemplary control patient **(B)** for the RPVI and LPVI region of 5 mm width as well as the respective margins of 10 mm width. The difference in median LI_upEn_ between the RPVI region and the RPVI margin as well as the LPVI region and the LPVI margin is summarized for all PVI patients **(C)** and all control patients **(D)** for different widths of the PVI regions and the margins in the lower panels. Colors indicate the elevation of LI_upEn_ within the margin compared to the PVI line with negative or small elevations in blue and large elevations in yellow. The black crosses mark the widths of 5 and 10 mm for PVI region and PVI margin as chosen for the exemplary distributions in **(A)** and **(B)**. Asterisks (*) denote statistically significant difference between distributions. LI, local impedance; RPVI, right pulmonary vein isolation; LPVI, left pulmonary vein isolation; PVI, plumonary vein isolation.

Figure 6A shows the extension of the analysis to an entire PVI lesion of an exemplary patient for an assumed PVI width of 5 mm and a margin of 10 mm width. For both right PVI (RPVI) and left PVI (LPVI), LI_upEn_ within the margin significantly exceeded LI_upEn_ within the PVI region. In contrast, the distributions of LI_upEn_ did not differ significantly for an exemplary control patient as shown in Figure 6B. Varying the width of the PVI region and the margin and averaging over all patients with previous PVI confirmed the observation. As reflected by positive values of ΔLI in Figure 6C, the PVI margin exceeded the PVI region in LI across all patients. Small PVI widths in combination with small margin widths did not show significant differences presumably because both the PVI region and the margin were embedded in the actual lesion. Larger widths consistently resulted in significant differences. In contrast, this trend did not appear for the average control patient as presented in Figure 6D. For all combinations of PVI widths and margin widths, ΔLI of the PVI group exceeded ΔLI of the control group by 5.1 Ω on average. When considering only PVI widths greater than or equal to 5 mm as well as margin widths greater than or equal to 10 mm, the ΔLI within the PVI group exceeded ΔLI within the control group by 7.7 Ω on average.

#### 3.4.4. LI in Regions of Native Pathological Substrate

**Figure 7:**
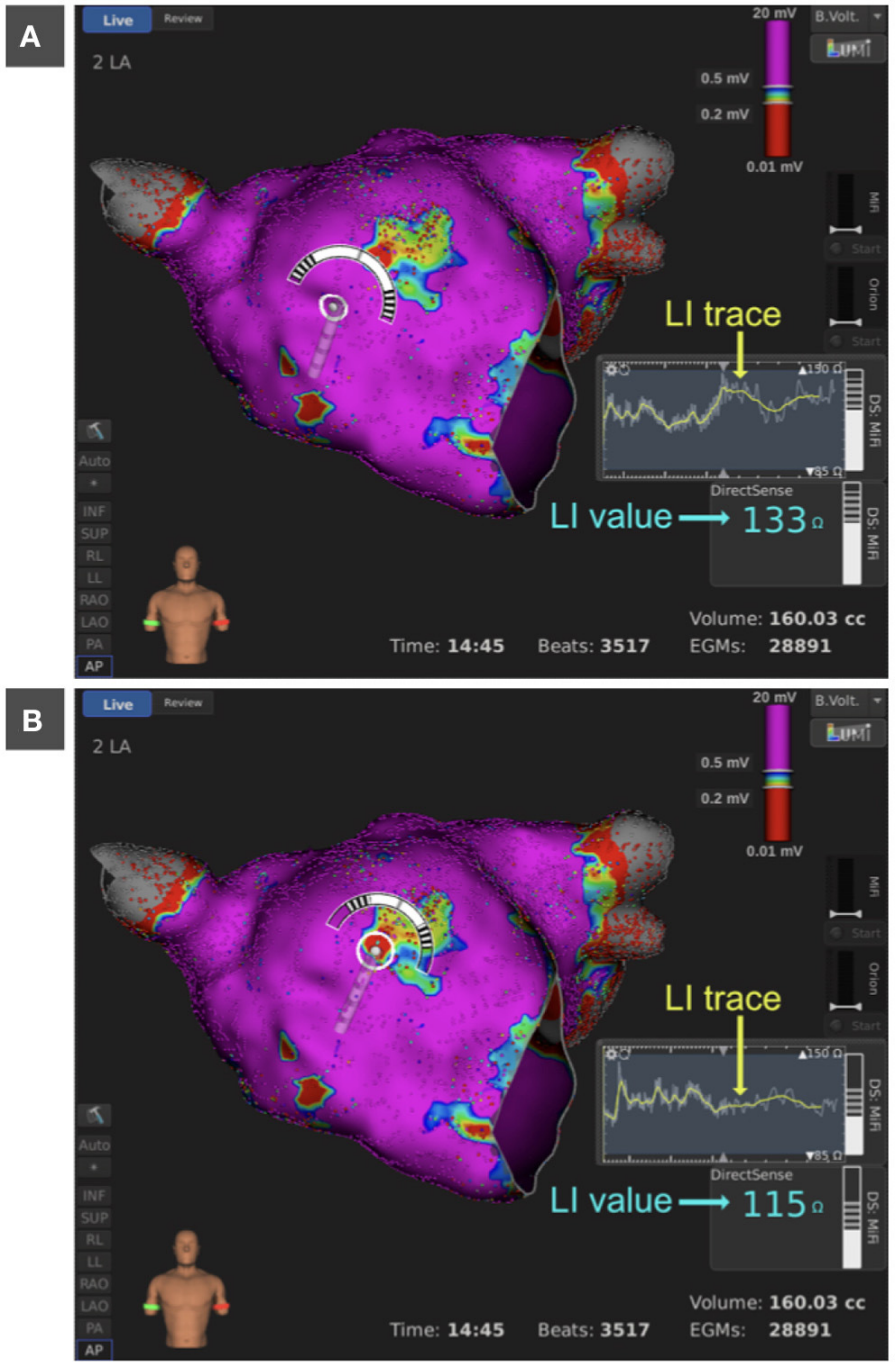
Bipolar voltage map of the LA in anterior view. Bipolar voltages below 0.2 mV are colored in red, above 0.5 mV in purple and between 0.2 and 0.5 mV in rainbow colors. The anterior wall presents centrally with a native pathological substrate of lowered bipolar electrogram amplitude. LI trace presented as an overlap of LI_raw_ (gray) and LI_movAvg_ (yellow) and LI value (light blue) within the pathological region **(A)** and the adjacent high voltage region **(B)**. LI, local impedance; LA, left atrium.

Besides unraveling previously ablated substrate, LI mapping was tested for its capabilities in distinguishing natively developed pathological substrate from healthy tissue. Since the ground truth of the pathological status of tissue was not available, an exemplary native scar at the anterior wall of the LA was deduced from thorough bipolar voltage mapping as displayed in Figure 7. The LI within the mapped scar area measured 115 Ω as compared to 133 Ω in the adjacent high voltage region. Figures 7A,B present screenshots of the respective measurements taken during the procedure with the LI trace in yellow and the LI value in light blue.

### 3.5. LI Oscillations

**Figure 8:**
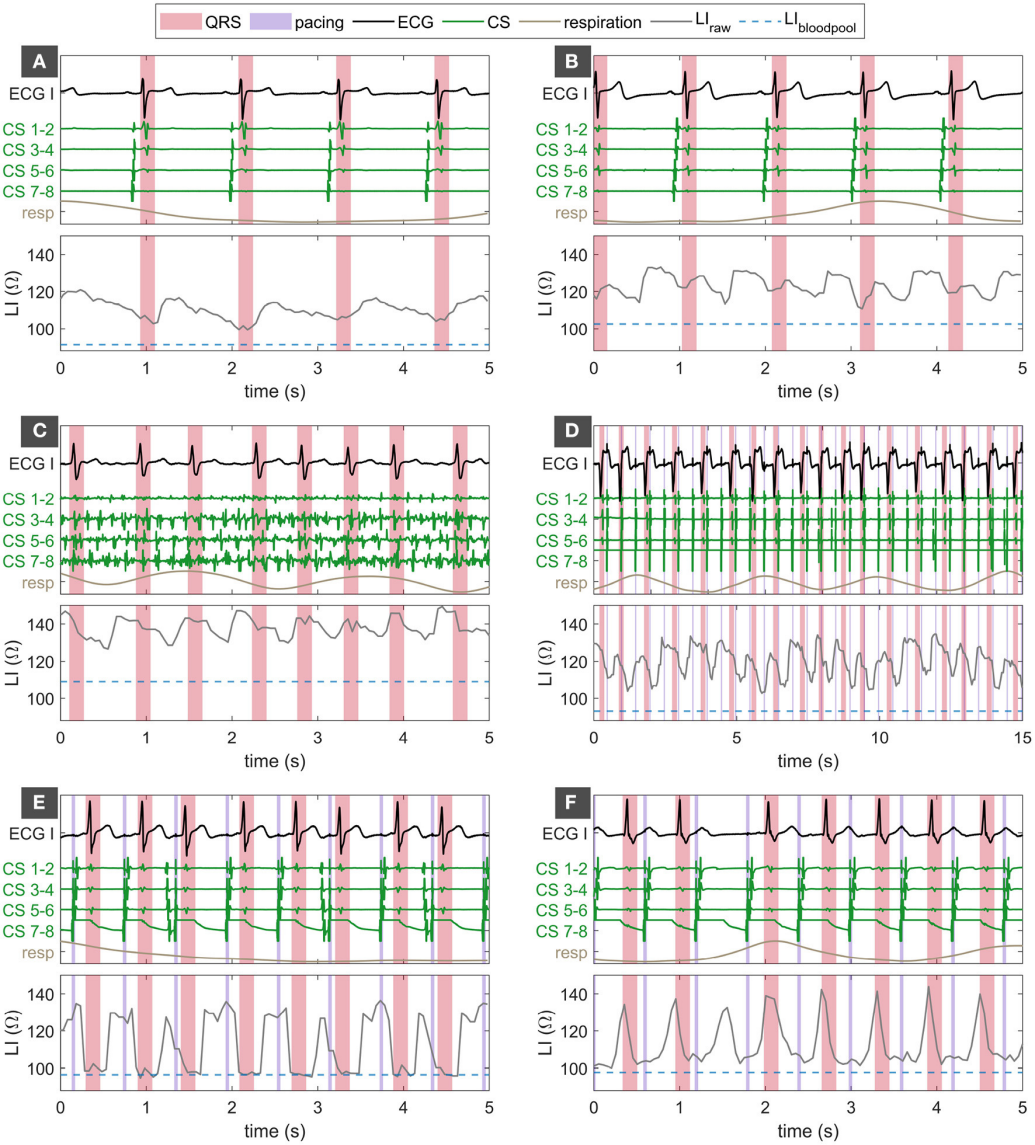
Panels **(A–F)** show different examples of oscillatory amplitudes, morphologies, and dependencies in LI_raw_. Traces of LI_raw_ in the respective lower panel in gray along with the corresponding bipolar CS electrograms in green, Einthoven lead I in black, and the respiratory phase in brown in the respective upper panel. LI_bloodpool_ is given as a broken blue line in the lower panel. QRS complexes are shaded in red. Pacing events are shaded in violet. LI, local impedance; ECG, surface electrocardiogram; ECG I, Einthoven lead I of the ECG; CS, coronay sinus.

LI_raw_ significantly oscillated predominantly with the atrial and ventricular rhythm as well as with the respiratory rate. Figure 8 depicts six exemplary LI traces in combination with Einthoven lead I and four bipolar CS electrograms. Based on the exemplary traces, the morphology, amplitude, and origin of oscillations in LI_raw_ were analyzed. Panels (A) and (B) in Figure 8 show acquisitions during sinus rhythm with moderate oscillatory amplitudes between 15 and 20 Ω. While the trace in Figure 8A comes with one distinctive maximum shortly after the ventricular contraction followed by a gradual decline in LI_raw_, the oscillatory morphology in Figure 8B is m-shaped and presents two maxima. Recorded in sinus rhythm, the oscillations could originate from the atrial contraction, the ventricular contraction or a superposition of both. Figure 8C displays a recording during AFib with comparable oscillatory amplitude. In view of the highly irregular atrial rhythm, the oscillations clearly relate to the ventricular contraction in this case with one oscillatory cycle per RR interval. The examples in Figures 8D–F were recorded during fixed rate pacing from the CS. Figure 8D covers an extended time window of 15 s and contains 3.5 respiratory cycles resulting in a superposition of cardiac and respiratory oscillations in LI_raw_. Figures 8E,F demonstrate the influence of atrial activation on the oscillatory components in LI_raw_. Physiologically, captured atrial stimuli are characterized by a proximal to distal CS sequence and a constant stimulus to CS interval. The third, sixth, and eighth stimulus in Figure 8E are preceded by a spontaneous atrial activation, indicated by the distal to proximal CS sequence followed by a loss of capture of the following stimulus. The difference in LI oscillation of the third, sixth, and eighth beat compared to the other beats suggests a dependency on the atrial activation sequence and the atrial-ventricular timing. A similar conclusion was drawn from the trace in Figure 8F. One of the atrial activations is not followed by a ventricular contraction. However, the oscillatory morphology in LI_raw_ is very similar to the other atrial beats that are followed by a ventricular activation being suggestive of an oscillatory origin in the atria.

**Figure 9:**
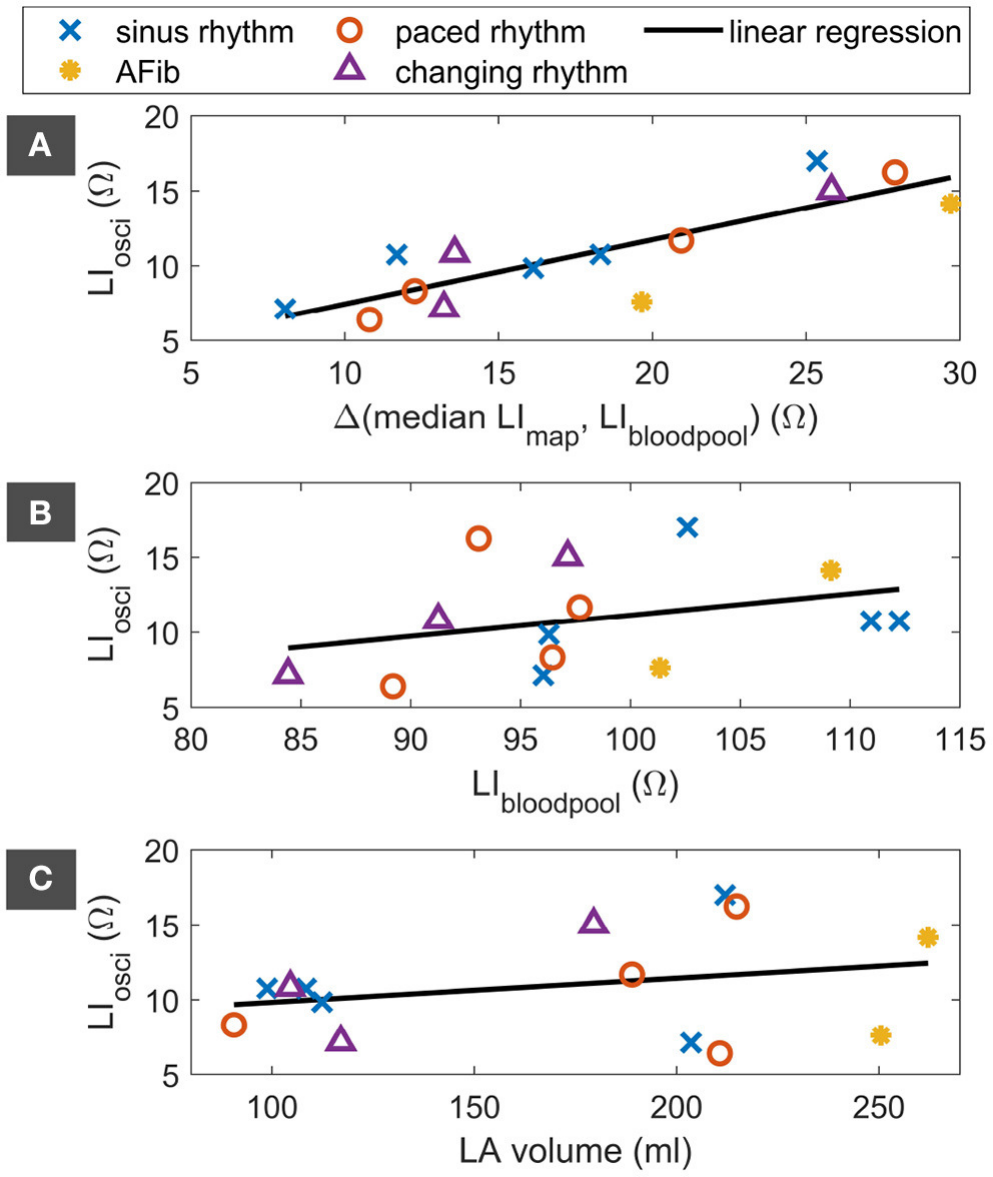
Dependency of the median LI oscillation on the elevation of the median LI_upEn_ of the LI map above LI_bloodpool_ **(A)**, on LI_bloodpool_ **(B)**, and on the LA volume **(C)**. Markers indicate the cardiac rhythm during map acquisition. The linear regression is displayed as black line. LI, local impedance; AFib, atrial fibrillation; LA, left atrium.

Figure 9 visualizes possible dependencies of the median oscillatory amplitude. The differences in the median oscillatory amplitude could not be explained by LI_bloodpool_ or the LA volume as indicated in Figures 9B,C as well as coefficients of determination R^2^ = 10.9 % and R^2^ = 7.6 %, respectively. The median LI oscillation amplitude correlated well with the elevation of the median LI_upEn_ above LI_bloodpool_ within the mapping region as displayed in Figure 9A and emphasized by a coefficient of determination R^2^ = 72.1 %. The median oscillatory amplitude was not dependent on the cardiac rhythm during map acquisition.

Causal relationships were neither found between the morphology of the oscillation and the anatomical region nor between the oscillation amplitude and the anatomical region.

## 4. Discussion

In summary, this study provided novel insights into the potential of LI mapping for substrate characterization. With the automatic determination of the bloodpool reference LI, the proposal of adequate preprocessing steps, and the assessment of LI as a surrogate for substrate characteristics, we integrally shed light on a previously unexplored field.

With the identification of native fibrotic and preablated scar tissue in previous PVI lesion areas by LI, substrate mapping by means of LI was proven possible and fosters confidence for the future of propagation independent substrate mapping. Figure 5 clearly shows the difference in information of voltage maps and LI maps: An area behind a closed ablation line does not show any voltage, but still the LI can be measured and used for substrate characterization. Despite the proof of concept presented in this work, the capabilities of LI-based substrate mapping are not yet fully exploited. Many confounding factors such as the distance to the endocardial wall, oscillations in LI_raw_ due to the cyclic loss of wall contact, varying contact force between catheter and endocardium, the orientation of the catheter, and partial coverage of the catheter by the sheath hinder the LI measurement. The distance to the endocardial wall, for example, was calculated based on the dynamic catheter position and the static endocardial shell. Cardiac movements caused by cardiac contractions and respiration introduce uncertainties in the distance that can only partially be accounted for. On average, the dependency of LI on the distance to the endocardium showed the same trend as previously observed in a controlled environment (Sulkin et al., 2018). The dependency is also in accord with the previously found correlation between LI and electrogram amplitude (Martin et al., 2018). If several LI measurements mapped to the same surface vertex, choosing the upper quartile meant to decide for those measurements with better endocardial contact in this work. However, the IntellaNav MiFi^TM^ OI comes without contact force sensor resulting in LI measurements at uncontrolled endocardial contact. A more accurate distance or contact measure has the potential to improve the accuracy of LI mapping in the future. The introduction of a contact force sensor within the IntellaNav Stablepoint^TM^ (Boston Scientific, Malborough, MA, USA) has been a recent step toward this direction. Many of the previously mentioned confounding factors could potentially also be resolved in the future by complementing the currently available measurements by multi-electrode injection and measurement circuits or by current injections at different frequencies.

LI mapping of 14 patients gave rise to the conclusion that the anatomical location of acquisition outside the PVs does not significantly bias the meaning of LI to conclude on substrate properties. The anterior, posterior, inferior, and septal wall showed similar but statistically different distributions of LI. Solely the posterior wall stood slightly out in absolute numbers with an increased LI, which could potentially be caused by the difference in catheter positioning. Depending on the location of the transseptal puncture, the catheter might reach the posterior wall with a by default increased contact force (Makimoto et al., 2014) resulting in a deeper immersion of the catheter tip into the atrial tissue compared to the remaining atrium. In contrast, the LI inside the PVs exceeded the LI in the remaining atrium presumably due to the smaller lumen and different substrate of the PVs as previously shown in impedance studies with generator impedance setups (Vaseghi et al., 2005). With the inferior PVs usually being smaller in diameter than the superior PVs (Dong et al., 2019), the smaller lumen could also explain higher LI values in the inferior PVs compared to the superior PVs.

The range of the automatically determined reference LI_bloodpool_ with a median slightly below 100 Ω compared well with other studies (Martin et al., 2018; Gunawardene et al., 2019).

Considering that standard treatments such as stand-alone PVI remain unsuccessful for a non-negligible subgroup of AFib patients (Tilz et al., 2012), substrate mapping has generally gained attention in recent years. While the most accurate picture of the substrate could be obtained from histological analysis, in-human histology is not possible to the required extent and one has to fall back upon rather indirect measures reflecting the composition and pathology of the substrate. Electrogram-based substrate mapping approaches such as voltage or fractionation mapping have led to ambiguous outcomes in various studies (Verma et al., 2010, 2015; Vogler et al., 2015). To a certain extent, electrograms indirectly reflect the state of the cardiac tissue by means of its effect on excitation propagation. However, it has to be taken into account that the direction of the propagation mechanism during mapping takes strong influence on electrogram morphology and amplitude. Impedance mapping, on the contrary, is mostly independent from the current mechanism of excitation. While histology cannot be performed, impedance mapping may bridge the gap by complementing existing electrogram-based substrate mapping approaches. Information on the resistivity of the tissue is not equivalent to histological analysis but can partially untangle the unknown composition of the underlying substrate. In combination with electrogram-based information on how the substrate supports and influences the spread of excitation, pathological areas can be explored from two different perspectives to build up an extensive picture of the atrial substrate. A thorough analysis of the complementarity of electrogram-based substrate mapping approaches and LI maps in a large patient population was out of the scope of this work but will need to bring further insights in future studies.

Many electroanatomical mapping systems are capable of measuring transthoracic generator impedance but do not yet incorporate high-frequency current injection and voltage measurement in an intra-cardiac catheter without including a cutaneous reference patch in the injection circuit. Local current injection and voltage measurement bears the advantage of pronouncing local influences on the spread of the electrical field with a decreased sensitivity to distant changes in conductivity caused by the respiratory cycle, e.g., While LI measurements are theoretically possible with any multi-electrode catheter, the Rhythmia HDx system with the IntellaNav MiFi^TM^ OI and the IntellaNav StablePoint^TM^ catheter are currently the only clinically available systems providing the hardware, software, and visualization tools to conduct in-human LI measurements. Therefore, the suggested pipeline for LI mapping can so far only be applied to Rhythmia HDx recordings with either the IntellaNav MiFi^TM^ OI or the IntellaNav Stablepoint^TM^ catheter.

The high correlation between the oscillatory amplitude and the elevation of the median mapping LI above LI_bloodpool_ within the mapping region strongly suggests that the common origin of oscillations in LI_raw_ is the establishment and the loss of endocardial contact. Since patients present with a wide variety of LI_bloodpool_ and presumably also a variety in the LI of the atrial tissue, the elevation of the median mapping LI above LI_bloodpool_ is a patient-specific determinant of the oscillatory amplitude.

Within an individual patient, the raw LI recording oscillated with different amplitudes, morphologies, and phases. Evidence was provided that the interaction of three oscillatory components—namely the atrial, the ventricular, and the respiratory component—led to the overall oscillation pattern observed in LI_raw_. The complex dynamics of the beating heart inside the breathing thorax suggests that the overall oscillation pattern is additionally dependent on a multifactorial chain of dependencies. The mechanical positioning of the catheter within the LA may play a significant role. With the sheath passing the atrial septum, the catheter will move with the septum to a certain extent. Furthermore, the curvature of the catheter within the LA can influence the actual catheter movement and stability. Additionally, different anatomical regions may move more during the atrial and ventricular beat than others. The complex interplay of the atrial movement with aforementioned mechanical factors could explain that no direct dependency was evident between the anatomical region and the oscillation amplitude within individual patients. While the atrial, the ventricular, and the respiratory component of LI oscillations were unraveled in this study, available measurements did not allow to conclude on the origin of additional oscillatory morphology and amplitude components, possibly due to unknown factors such as the curvature of the catheter and the complex dynamics of cardiac mechanics.

With the moving average and the upper envelope, two different methods were described to tackle the oscillations in LI_raw_ with a high potential to flaw the meaning of LI maps. Coming from the insight that oscillations originate from the establishment and loss of wall contact, the summits of one oscillatory cycle were assumed to best resemble the LI of the underlying tissue. While the moving average takes into account any value during an oscillatory cycle, the upper envelope focuses on the interpolation of the maxima and, therefore, maximizes the elevation above the baseline LI_bloodpool_. Therefore, most analyses in this work favored LI_upEn_ over LI_movAvg_ despite the current implementation of the moving average in the clinical system. A potential drawback of LI_upEn_ compared to LI_movAvg_ is the increased impact of high amplitude artifacts. However, conservative exclusion criteria led to the conclusion that the advantages of LI_upEn_ prevail.

### 4.1. Limitations

While this study showed that LI mapping bears great potential, the limitations as discussed in the previous paragraphs will be summarized in the following: Despite the proposal of adequate preprocessing steps, confounding factors remain and hinder LI mapping. Above all, the lack of a wall contact or contact force sensor results in uncertainties that should be addressed in future work. Since this study focused on the exploration of LI mapping itself, a thorough comparison to other substrate mapping approaches based on electrograms was out of the scope of this work. The complementarity of different substrate mapping approaches remains to be quantified in future analyses. Finally, the described LI mapping methods are so far only applicable to the IntellaNav MiFi^TM^ OI and the IntellaNav Stablepoint^TM^ being the only clinically available catheters equipped with LI measurement circuits.

### 4.2. Conclusion

To conclude, this study demonstrated that LI mapping shows great potential to complement electrogram-based substrate mapping. Both previously ablated and native scar areas were identified irrespective of local excitation in LI recordings. Oscillations pose a major challenge to adequate preprocessing. The upper envelope of the raw LI measurement should be preferred to the moving average in order to suppress oscillation artifacts while maximizing the elevation above the baseline LI of the surrounding bloodpool.

## Data Availability Statement

The raw data supporting the conclusions of this article will be made available by the authors upon reasonable request, without undue reservation.

## Ethics Statement

The studies involving human participants were reviewed and approved by the Ethics Committee Freiburg. The patients/participants provided their written informed consent to participate in this study.

## Author Contributions

LU, OD, and AL contributed to the conception of the study. Data was recorded by AL and KS. LU, LS, and MS conducted the analyses. Results were discussed and interpreted by LU, TO, AH, OD, and AL. LU wrote the first draft of the manuscript. LU, TO, AH, OD, and AL thoroughly revised the manuscript. All authors contributed to manuscript revision, read, and approved the submitted version.

## Funding

We gratefully acknowledge financial support by the Deutsche Forschungsgemeinschaft (DFG, German Research Foundation) Project-ID 394433254 (LU 2294/1-1, DO 637/23-1) and funding from the European Union's Horizon 2020 research and innovation programme under the Marie Skłodowska-Curie grant agreement No 860974. We are thankful for support by Boston Scientific in establishing the clinical part of the study and acknowledge support by the KIT-Publication Fund of the Karlsruhe Institute of Technology.

## Conflict of Interest

TO is employed by Boston Scientific, Ratingen, Germany. AL is a consultant for Boston Scientific and has received speakers-fees from Boston Scientific, Biosense Webster and Bristol-Myers Squibb. The remaining authors declare that the research was conducted in the absence of any commercial or financial relationships that could be construed as a potential conflict of interest.

## Publisher's Note

All claims expressed in this article are solely those of the authors and do not necessarily represent those of their affiliated organizations, or those of the publisher, the editors and the reviewers. Any product that may be evaluated in this article, or claim that may be made by its manufacturer, is not guaranteed or endorsed by the publisher.
